# *Caenorhabditis elegans* respond to high-glucose diets through a network of stress-responsive transcription factors

**DOI:** 10.1371/journal.pone.0199888

**Published:** 2018-07-10

**Authors:** Jonathan Alcántar-Fernández, Rosa E. Navarro, Ana María Salazar-Martínez, Martha Elva Pérez-Andrade, Juan Miranda-Ríos

**Affiliations:** 1 Programa de Doctorado en Ciencias Biomédicas, Universidad Nacional Autónoma de México (UNAM), México, Ciudad de México, México; 2 Unidad de Genética de la Nutrición, Depto. de Biología Molecular y Biotecnología, Instituto de Investigaciones Biomédicas, UNAM e Instituto Nacional de Pediatría, México, Ciudad de México, México; 3 Departamento de Biología Celular y Desarrollo, Instituto de Fisiología Celular, Universidad Nacional Autónoma de México, Ciudad de México, México; 4 Departamento de Medicina Genómica y Toxicología Ambiental, Instituto de Investigaciones Biomédicas, Universidad Nacional Autónoma de México, Ciudad de México, México; INSERM U869, FRANCE

## Abstract

High-glycemic-index diets, as well as a sedentary lifestyle are considered as determinant factors for the development of obesity, type 2 diabetes, and cardiovascular diseases in humans. These diets have been shown to shorten the life span of *C*. *elegans* in a manner that is dependent on insulin signaling, but the participation of other signaling pathways have not been addressed. In this study, we have determined that worms fed with high-glucose diets show alterations in glucose content and uptake, triglyceride content, body size, number of eggs laid, egg-laying defects, and signs of oxidative stress and accelerated aging. Additionally, we analyzed the participation of different key regulators of carbohydrate and lipid metabolism, oxidative stress and longevity such as SKN-1/NRF2, HIF-1/HIF1α, SBP-1/SREBP, CRH-1/CREB, CEP-1/p53, and DAF-16/FOXO, in the reduction of lifespan in glucose-fed worms.

## Introduction

Obesity is predicted to impact the health of more than 1 billion people by the year 2030 [1]. High-glycemic-index diets in industrialized and emerging countries include a large proportion of processed carbohydrates or sugars, which are readily metabolized to glucose, causing a fast increase in blood glucose level, and altering glucose homeostasis, influencing in a negative way the development, fertility, and lifespan in organisms as diverse as yeasts, worms, and mammals [2]. In particular, high-glucose diets (HGD) are known to generate reactive oxygen species (ROS), that include radical and non-radical oxygen species such as hydroxyl radical (HO.), superoxide anion (O_2_^-^), and hydrogen peroxide (H_2_O_2_), which can damage lipids, proteins and nucleic acids and could lead to cell death [3]. In this context, oxidative stress has been suggested as a factor related to the development of several diseases, including obesity and diabetes [4, 5].

The worm *Caenorhabditis elegans* has been used as a model organism in many areas of biomedical research [6, 7]. For example, as there is a high homology between human and *C*. *elegans* genes, this worm has been used as a model of human diseases [8, 9]. Furthermore, *C*. *elegans* has been used in studies of glucose-induced toxicity [2, 10–12]. These reports have documented that HGD affect growth, fertility, aging and lifespan. Nonetheless, these studies have been performed under a wide range of experimental conditions, i.e. different glucose concentrations used, diverse administration regimes, and varied times of exposure, making it difficult to compare the results obtained.

It has been established that a shortened lifespan in glucose-fed worms is in part due to the activation of the insulin/IGF-1 signaling (IIS) pathway [11]. The IIS network can be summarized as follows (reviewed in [13]): insulin like-peptides bind to their receptor (DAF-2), recruiting an insulin receptor substrate. This activates the phosphatidyl inositol-3-OH kinase AGE-1/PI3K, increasing the level of phosphatidyl inositol (3,4,5)-triphosphate (PIP3), which in turn activates a kinase cascade, formed by 3-phosphoinositide-dependent protein kinase 1 (PDK-1), protein kinase B (AKT-1/-2), and serum- and glucocorticoid-inducible kinase-1 (SGK-1). This cascade inactivates by phosphorylation the transcription factor DAF-16/FOXO, resulting in the translocation of DAF-16/FOXO from the nucleus to the cytosol. AMP-activated protein kinase (AMPK/AAK-2) is also able to phosphorylate DAF-16/FOXO. AKT-1/-2 is also involved in the sequestration in the cytosol of the oxidative stress-responsive NRF transcription factor SKN-1. Although much is known about the activation of the IIS pathway in response to HGD, the involvement of other signaling pathways has not been thoroughly considered.

In the present study, we evaluated biochemical (glucose uptake, glucose and triglyceride content), as well as physiological (body size, lifespan, number of eggs laid, and egg-laying defects) changes experienced by worms exposed from the L1 to the L4 larval stage to increasing concentrations of glucose in the growth medium. We present evidence that worms fed a HGD face oxidative stress and induce enzymatic and non-enzymatic antioxidant systems so that the organism can cope with the imposed stress. Additionally, we assessed the participation of transcriptional regulators that are known to be involved in longevity, lipid accumulation and oxidative stress, such as SKN-1/NRF2, HIF-1/HIF1α, SBP-1/SREBP, CRH-1/CREB, CEP-1/p53, and DAF-16/FOXO [14–19], that could be involved in the genetic mechanisms by which glucose reduces lifespan, by studying the effect that mutants or RNAi knock-downs have on the lifespan of glucose-fed worms, and the changes that dietary glucose induce in the mRNA accumulation of the above mentioned transcription factors.

## Materials and methods

### Strains and culture conditions

The wild-type Bristol (N2), CF1553 *muls84* [(pAD76) *Psod-3*::GFP+*rol-6(su1006)*], GR1307 *daf-16(mgDf50)* I, CF1038 *daf-16(mu86)* I, TJ1 *cep-1(gk138)* I, EU84 *unc-5(e53) skn-1(zu67) IV/nT1* [*let-*?*(m435)*] (IV;V) strains were cultured using NGM plates seeded with *E*. *coli* OP50-1 and raised at 18°C, as previously described [20]. The worms were synchronized with alkaline hypochlorite solution [21], a condition in which only eggs can survive, and eggs were washed with M9 buffer solution. After synchronization, worms were seeded on a NGM plate (control condition) and in glucose-supplemented plates, and fed with *E*. *coli* OP50-1 until they reached L4 larval stage. Glucose (Sigma) was added to the mix of agar and salts of the NGM medium in order to obtain 20, 40, 80, or 100 mM glucose concentration. The *E*. *coli* HT115(DE3) strain was used for the feeding RNAi experiments.

### Preparation of worm lysates

L4 stage worms were harvested and washed three times with M9 buffer (42.26 mM Na_2_HPO_4,_ 22.04 mM KH_2_PO_4,_ 85.56 mM NaCl, and 0.87 mM MgSO_4_) in order to remove all bacteria. After removing as much as possible of M9 buffer, worms were resuspended in lysis buffer: 50 mM HEPES, 50 mM KCl, 1mM EDTA, 1mM EGTA, 5 mM phosphate β-glycerol, 0.1% (v/v) Triton X-100, 50 mM sodium fluoride, 1 mM sodium orthovanadate, 5 mM sodium pyrophosphate, 0.2 mM phenylmethanesulfonylfluoride and protease inhibitor (Complete, Roche). Worms were frozen in liquid nitrogen and thawed at 37°C for three times, then worms were sonicated in ice with an ultrasonic processor unit (Sonics®) in three cycles of 30 sec. (Amplitude = 60, pulse = 3) for 1 min, and centrifuged at 12000 rpm at 4°C for 15 min. The supernatant was collected and stored at -70°C. An aliquot was used for protein quantification by Bradford assay (BioRad Protein Assay).

### Glucose and triglyceride content measurement

Freshly-made lysates of worms from the different growth conditions were prepared, and internal glucose and triglyceride content was determined by applying the same protein amount for each test. For glucose quantification, the Reflotron Plus/Sprint System (Roche) was used, which is based in the enzymatic oxidation of glucose to give δ-D-gluconolactona plus hydrogen peroxide; the hydrogen peroxide then changes the color of a chromogen which is quantitated by reflectance photometry (http://www.cobas.com/home/product/point-of-care-testing/reflotron-plus-sprint-system.html). For triglyceride quantification, we used a colorimetric assay in which the glycerol from the triglycerides is enzymatically converted first to glycerol-3-phosphate and then to dihydroxyacetone phosphate plus hydrogen peroxide. The hydrogen peroxide then enzymatically reacts with 4-aminofenazone and p-clorofenol to give quinone, which is spectrophotomectrically determined at 505 nm using the SPINREACT system (Spinreact, Sant Esteve de Bas, Spain; www.spinreact.com).

### Glucose uptake assay

Glucose uptake was determined by using the 2-deoxy-D-glucose (2DG) Uptake Measurement kit (Cosmo Bio CSR-OKP-PMG-K01E), according to the manufacturer’s recommendations. In short, L4 stage larvae were incubated in 0.5 mM 2-DG (Sigma D8375) for 2 h. Then, larvae were washed three times with cold M9 buffer, and larvae were weighted as a way to normalize the quantification. Larvae were lysed with extraction buffer (10 mM Tris-HCl pH 8.1), according to [22]. Then, 80 μl of extraction buffer was added to each sample, frozen with liquid nitrogen and thawed at 37°C, then samples were heated at 85°C for 40 min. Samples were centrifuged at 13000 rpm at 4°C for 20 min, the supernatant was collected in a new tube and stored at -20°C. As a positive control, larvae grown in the absence of glucose were incubated in 0.5 mM of 2DG plus insulin at 80 mU/mL for 2 h, and processed in the same way as described above. Recombinant human insulin was administered (Humulin, 100 UI/mL, Lilly). Insulin concentration used was taken from a previous report [23].

### Body length and area measurement

Fifty L4 larvae from each condition (glucose 0, 20, 40, 80 or 100 mM) were picked randomly from unlabeled plates. Experiments were performed in triplicate. Worms were photographed under a 20X objective in the Nikon Optiphot-2 microscope. Images were analyzed using the Image J software [24] (http://imagej.nih.gov/ij/). Lengths were traced from the tip of the head to the tip of the tail and areas were obtained by delineating all of the body. Calibration measurement was made with a stage micrometer (OBM1/100).

### Determination of the number of eggs laid

To determine the number of eggs laid, 30 L4 worms were grown on NGM or NGM supplemented with 20, 40, 80 or 100 mM glucose. In the next three days worms were transferred to new plates (either control or glucose-supplemented as specified) and L1 larvae were scored for each plate. Then, 30 L4 larvae from the P0 were transferred to new NGM plates (either control or glucose-supplemented as specified) for the F1 generation. This process was replicated for the F2 generations.

### Determination of egg retention with internal hatching or “bagging”

One hundred L4 stage control or glucose-fed worms were seeded in NGM plus 20, 40, 80 or 100 mM glucose in triplicates, and the occurrence of “bagging” was monitored for 10 days using a stereoscopic microscope (Nikon SMZ800), as reported previously [25].

### Determination of Aspartate transaminase (AST) and alkaline phosphatase (ALP) enzyme activities

AST and ALP enzyme activities were assayed in L4 larvae homogenates by colorimetric assays (Spinreact, Sant Esteve de Bas, Spain; www.spinreact.com), following manufacturer’s instructions. Enzyme activities were adjusted by the protein content in the sample.

### Lipid peroxidation assessment

Lipid peroxidation was quantified on L4 larvae by assaying malondialdehyde (MDA), which was determined by the thiobarbituric acid (TBA) method [26]. Briefly, freshly-made lysates of L4 larvae were incubated in TBA (0.375%) dissolved in TCA (30%), heated by boiling for 45 min, kept in ice for 15 min, followed by a centrifugation at 1000×g for 10 min. Supernatants were read at 532 nm on a spectrophotometer. MDA content was calculated against a standard curve of tetramethoxypropane (TMPO) (Sigma 108383–100). The MDA content was adjusted by the protein content in the sample. As a positive control for lipid peroxidation induction, normally fed worms were incubated in 0.2 mM paraquat (Sigma Aldrich 36541) for 1 h at room temperature, samples were processed in the same way for every determination.

### Measurement of mitochondrial superoxide dismutase and catalase enzyme activities

For quantification of mitochondrial SOD activity, the mitochondrial fraction was isolated from L4 larvae grown at the appropriate glucose concentration. Mitochondrial isolation was done as reported by [27]. mtSOD activity was measured using a commercial SOD determination kit (Sigma Aldrich, 19160), according to the manufacturer's recommendations. Catalase activity was measured according to [28] with slight modifications as follows: worms were homogenized in phosphate buffer (0.1 M, pH 7.0), then protease inhibitor (Complete, Roche) was added. After protein was quantified by the Bradford method, 20 μg of total protein were incubated for 30 s in phosphate buffer (0.1 M, pH 7.0, 12 mM H_2_O_2_). Reaction was stopped by addition of ammonium molibdate solution at 32.4 mM. Reactions were read at 405 nm, and the specific activity was calculated. As a positive control for mtSOD and catalase activities induction, control worms were incubated in 0.2 mM paraquat for 1 h at room temperature, samples were processed in the same way for every determination.

### *Psod-3*::GFP reporter strain expression and heat shock experiments

Approximately 200 L1 larvae from *Psod-3*::*gfp* synchronized animals were seeded on 60 mm plates of NGM media supplemented with 0, 20, 40, 80 or 100 mM glucose and incubated at 20°C until they reached L4 larval stage. At this stage plates were transferred to an air incubator at 31°C for 8 h as indicated [29]. After heat shock, animals were anesthetized with 10 mM tetramisole, mounted on agarose pads at 2% and observed on a Nikon Eclipse E600 microscope equipped with an AxioCam MRc camera and Zeiss AxioVision software. Quantification of GFP fluorescence was performed using Image J software as previously indicated [30].

### Determination of total glutathione, GSH and GSSG

For glutathione quantification, lysates and determination were made the same day. Glucose-fed and control L4 larvae were harvested and washed with M9 buffer three times. After removing as much as possible of M9 buffer, worms were resuspend in ice-cold metaphosphoric acid (5% w/v) (Sigma), then worms were sonicated in ice with an ultrasonic processor unit (Sonics) in three cycles of 30 s (Amplitude = 60, pulse = 3) for one minute and centrifuged at 12000 rpm at 4°C for 15 min. The supernatant was collected and kept on ice. Glutathione was quantified with HT Glutathione assay kit (Cat. 7511-100-K, Trevigen) according to the manufacturer´s recommendations. As a positive control for induction of oxidative stress, control worms were incubated in 0.2 mM paraquat for 1 h at room temperature, samples were processed in the same way for every determination.

### Lifespan assessment

Synchronized L1 worms were grown to young adults in NGM supplemented with 0 (control), 20, 40, 80 or 100 mM glucose until they reached the adult stage, then 100 worms from each condition were seeded on a new Petri dish containing the same amount of glucose that was previously used. We chose the number of 100 worms to be studied as with this number we have a probability of 0.82 to detect a change in lifespan at a significance level of 0.05, as calculated by a parametric survival-time model of *C*. *elegans* based on the Gompertz equation [31]. The experiment was conducted blind with the experimenter not knowing the identity of the treatment. Worms were counted and transferred daily to get rid of embryos and L1 larvae. Dead worms were counted on a daily basis. Worms showing egg-laying defects and worms fixed to the wall of the Petri dish were censored from counting. Lifespan assays were performed in triplicate.

### Feeding RNAi

Bacterial clones carrying RNAi feeder plasmids of *crh-1*, *hif-1* and *sbp-1* were obtained from the *C*. *elegans* RNAi v1.1 Feeding Library (Open Biosystems, Huntsville, AL, USA) [32]. Empty vector (pL4440) was used as a control. Each RNAi colony was grown overnight in Luria broth with 100 μg/ml ampicillin (Sigma-Aldrich) and 12.5 μg/ml tetracycline (Sigma-Aldrich). The next day, fresh Luria broth was seeded with these overnight cultures and grown at 37°C with constant shaking; 1 mM isopropylthiogalactoside (IPTG, Fermentas) was added to the culture to induce dsRNA expression for 2 hours. All of the RNAi clones used in this work were verified by DNA sequencing and subsequent BLAST analysis to confirm their identity.

### Lifespan determination on RNAi knock-down strains

L1 larvae were seeded on plates containing RNAi bacteria, then 0.4 mM IPTG was added, and larvae were allowed to develop to adults, and then they were synchronized. The F1 worms were seeded on new plates containing RNAi bacteria in the absence of glucose (control condition) or in 100 mM glucose, and allowed to develop to L4 stage. Approximately 120 L4 worms undergoing RNAi treatment were transferred to fresh RNAi agar plates for lifespan experiments. To ensure the continued efficacy of RNAi knock-down, animals were fed everyday with freshly induced RNAi bacteria. The rest of F1 worms were harvested to assess RNAi knockdown efficiency determination by real time PCR. Worms were counted and transferred daily to get rid of embryos and L1 larvae. Dead worms were scored on a daily basis, counting worms that do not respond to a gentle touch with a sterilized platinum wire. Worms that died of protruding/bursting vulva, bagging, or crawling off the agar were censored.

### Quantitative RT-PCR

Total RNA was purified from worms grown in all of the conditions tested using Trizol (Invitrogen), according to the manufacturer recommendations and further purified using RNase kit (Qiagen). cDNA was generated with 1 μg of total RNA in a 10 μl reaction using Revert Aid enzyme (Fermentas). Then, 10ng of cDNA was used as a template in qRT-PCR analysis, that was performed on a Step One Real Time PCR System (Applied Biosystems) using SYBR Green PCR Master Mix (Applied Biosystems), following the manufacturer’s instructions. Primers used are listed in S1 Table. Each qPCR reaction was performed using five biological replicates in triplicate each. PCR program was: 10 min at 95°C, 40 cycles of 95°C for 15 s, Tm (see S1 Table for each oligonucleotide pair) for 30 s and 70°C for 30 s. Specificity of primers was corroborated by electrophoresis in a polyacrylamide gel and by melting point analysis. Y45F10D.4 gene was used as a reference accordingly to [33]. The relative expression ratio of the mRNA relative to Y45F10D.4 mRNA expression was calculated as previously described [34].

### Statistical analysis

Descriptive statistics was performed in all data set. If normality failed, data were analyzed using a non-parametric test. Differences between groups were tested using one-way ANOVA (parametric test), followed either by a Newman-Keuls for multiple comparison or Kruskal Wallis (non-parametric test) followed by Dunn´s method. Lifespan assays were analyzed using the log-rank (Mantel-Cox) test followed by the Bonferroni method with the OASIS software. Number of eggs laid assays were analyzed using a two-way ANOVA. *P* values of less than 0.05 were regarded as statistically significant. Experimental data are shown as mean values for at least four independent assays, and SEM are indicated by error bars. Analyses were performed using GraphPad Prism 6.0 software.

## Results

### Glucose-fed worms showed an increased glucose content, glucose uptake, and triglyceride accumulation

**Fig 1:**
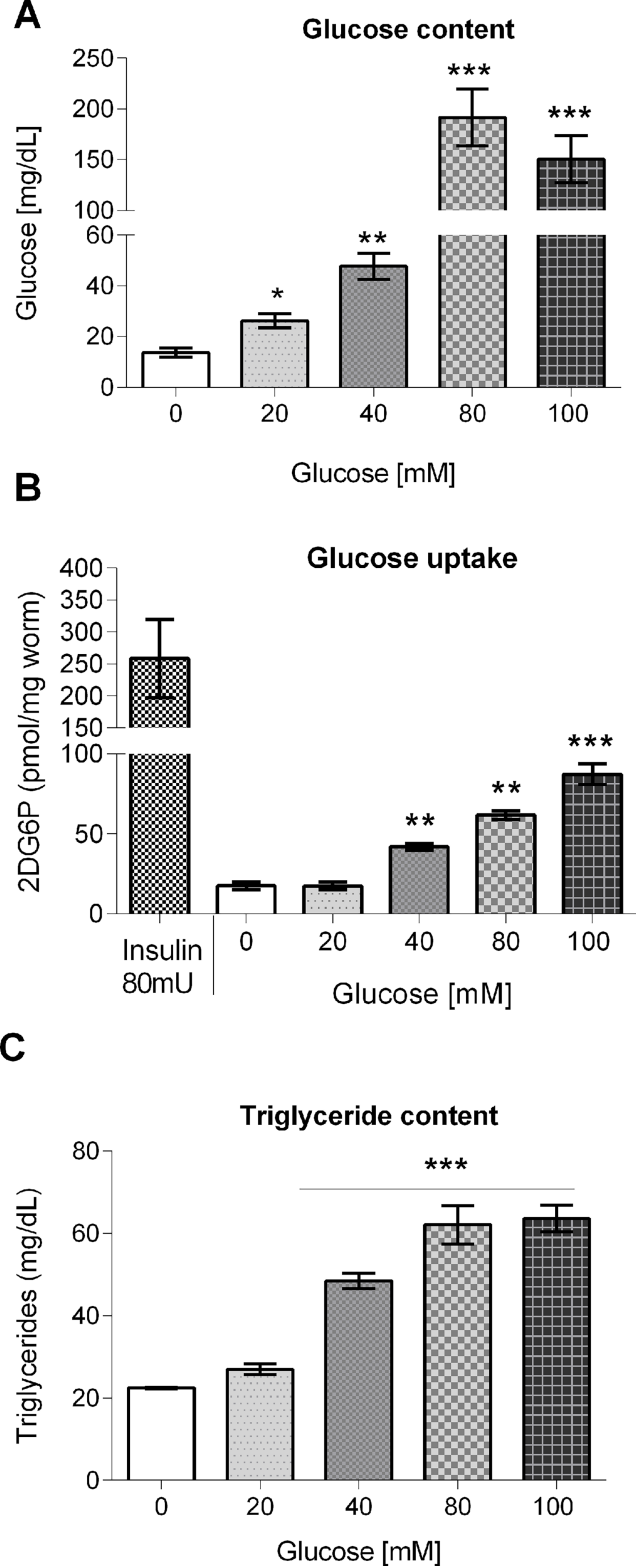
Glucose content, glucose uptake, and triglyceride content in glucose-fed worms. Worms were exposed from L1 to L4 larval stage to 20, 40, 80 or 100 mM glucose, and (A) glucose content, (B) glucose uptake, or (C) triglyceride content was determined. Values are expressed as median ± interquartile range (IQR) (n = 6).*P < 0.05, **P < 0.01 or ***P < 0.001 for the indicated comparison (calculated using the Kruskal-Wallis test).

We determined the accumulation of glucose in the body of worms that were grown in control medium or glucose-supplemented medium, and observed an increase of 2-, 3-, 14- or 11-fold in glucose content in worms grown at 20, 40, 80 or 100 mM glucose with respect to control (16.63 ± 1.82 mg/dL) (Fig 1A). Next, we determined glucose uptake using a 2-deoxy-D-glucose (2-DG) incorporation assay. We found that glucose uptake was increased by 2-, 4- or 7-fold compared to control (17.53 ± 2.29 nmol/mg worm) in worms exposed to 40, 80 or 100 mM glucose, respectively (Fig 1B). As a positive control, larvae that were grown in the absence of glucose was incubated for 2 h with insulin (80 mU/ml). Glucose uptake in insulin-treated larvae was increased as expected (Fig 1B). Furthermore, as glucose is a known precursor of triglycerides, we measured triglyceride accumulation in worms fed a HGD. We found that triglyceride content was augmented by 2-fold at 40 mM glucose, and 3-fold at 80 or 100 mM glucose (control = 22.48 ± 0.24 mg/dL) (Fig 1C). Together, these findings suggest that glucose supplemented into the growth medium is ingested by the worms, modulates its own uptake and is metabolized into triglycerides.

### Glucose-fed worms are thicker and longer than control worms

**Fig 2:**
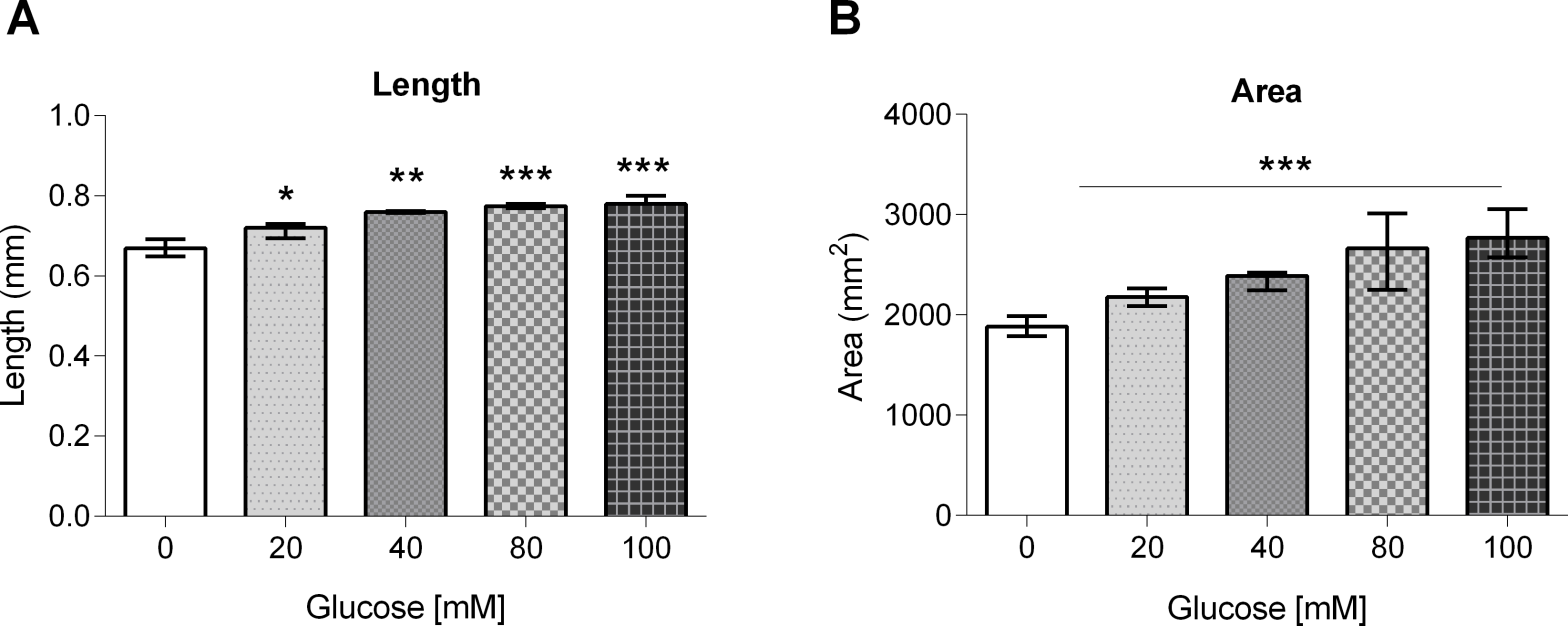
Body length and area of glucose-fed worms. Worms were exposed from L1 to L4 larval stage to 20, 40, 80 or 100 mM glucose, and their (A) length, or (B) area, was measured. Experiments were performed in triplicate. Values are expressed as mean ± SEM (n = 50). *P < 0.05, **P < 0.01 or ***P < 0.001 for the indicated comparison (calculated using a one-way ANOVA test).

As it has been reported that food limitation results in a reduced body size in *C*. *elegans* [35], we aimed to determine if growth in a HGD caused a change in body length and area. We found that worms grown at glucose 20, 40, 80 or 100 mM were 2, 13, 16 or 17% larger than control worms (0.67 ± 0.01 mm) (Fig 2A). As well, glucose-fed worms were thicker than control (1.92 ± 0.04 mm^2^), showing an increase in area of 13, 22, 36 or 44% at 20, 40, 80 or 100 mM glucose, respectively (Fig 2B). These data suggest that glucose consumption gives rise to worms that are longer and thicker than worms fed a diet without glucose added.

### Glucose exposure decreased the number of eggs laid and increased egg retention with internal hatching events (bagging)

**Fig 3:**
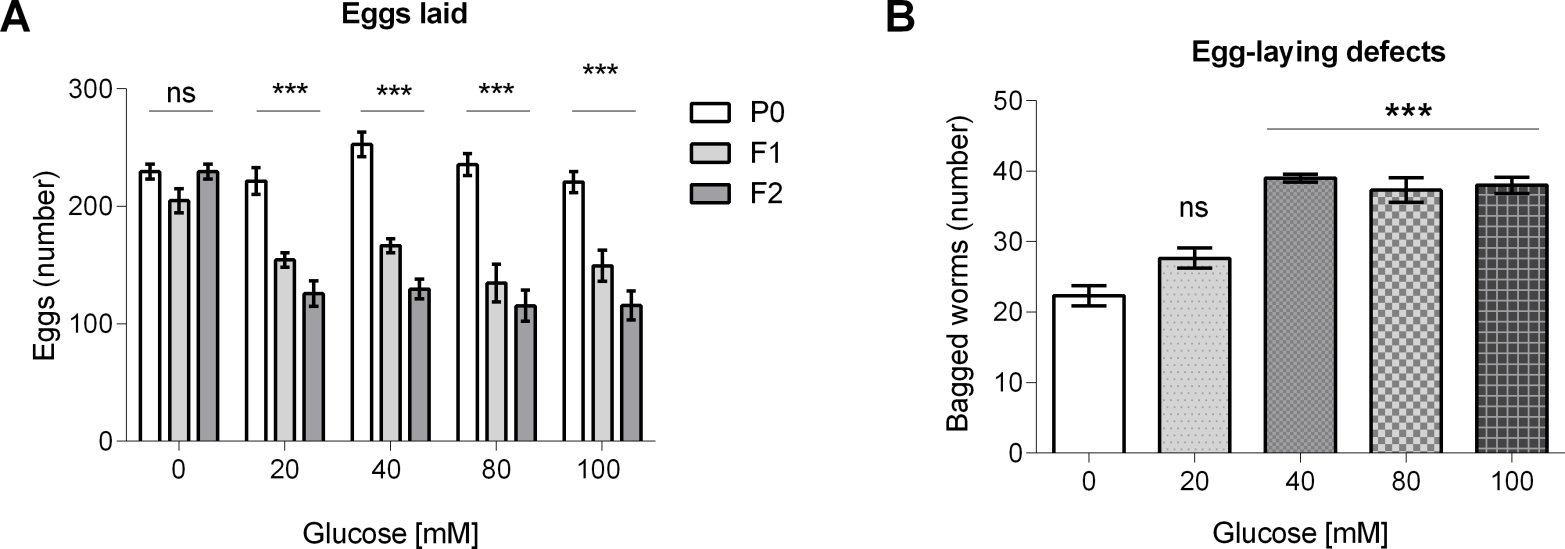
Number of eggs laid and egg retention with internal hatching frequency in glucose-fed worms. (A) Worms were exposed to 20, 40, 80 or 100 mM glucose for three generations (P0, F1 and F2) and the number of eggs laid in each generation was determined. (B) Worms were exposed to 20, 40, 80 or 100 mM glucose for only one generation and the frequency of egg retention with internal hatching or “bagging” was determined. Values are expressed as mean ± SEM (n = 100). *P < 0.05, **P < 0.01 or ***P < 0.001 for the indicated comparison (the number of eggs laid was analyzed using a two-way ANOVA test, while egg retention with internal hatching frequency was analyzed using a one-way ANOVA test).

As we have previously reported that starvation results in a reduced brood size [36], we asked if a HGD would have any impact in the progeny of glucose-fed worms. For this, we determined the number of eggs laid in the first three days post-L4 stage by worms that were grown from the L1 larval stage to adults for three generations (P0, F1, and F2) in a HGD. We found that worms from the P0 generation did not show any changes in the number of eggs laid (220 worms), but worms from the F1 and F2 generations showed a decrease of 25–39% or 45–50%, respectively (Fig 3A). As the number of eggs laid was decreased in glucose-fed worms, we hypothesized that this was probably due to defects in egg-laying capability. We determined the egg retention with internal hatching or “bagging” frequency at generation P0 of glucose-fed animals, as it has been reported that worms grown under stressful conditions showed problems in egg-laying as embryos hatch inside the worms and kill the mothers [25]. We observed an increase of 67–70% in “bagging” in worms fed a HGD with respect to control (20 worms showed “bagging”) (Fig 3B). Taken together, these data suggest that glucose altered the worm´s reproductive fitness, as glucose-fed worms showed a reduction in the number of eggs laid, partially as a consequence of an increase in “bagging”.

### Glucose exposure induced Aspartate Aminotransferase and Alkaline Phosphatase enzyme activities

**Fig 4:**
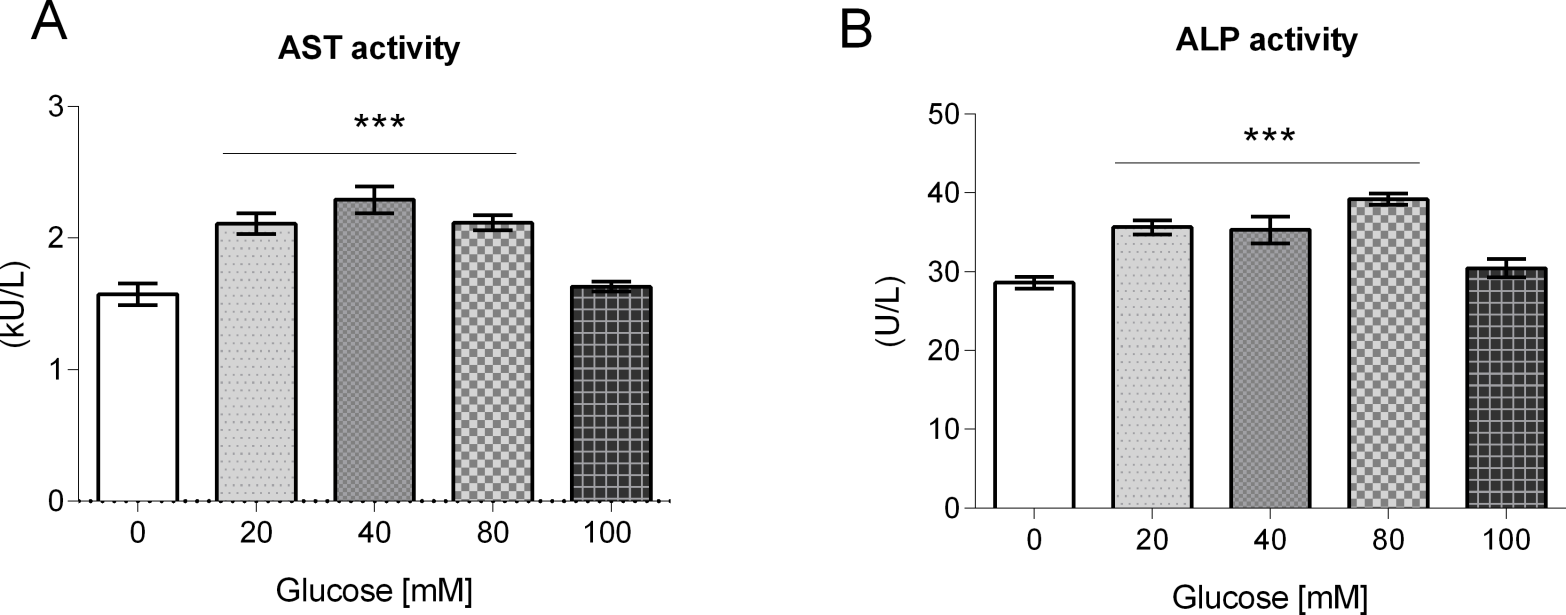
Aspartate Aminotransferase (AST) and Alkaline Phosphatase (ALP) enzyme activities in glucose-fed worms. Worms were exposed from L1 to L4 larval stage to 20, 40, 80 or 100 mM glucose, then (A) AST and (B) ALP enzyme activities were measured. Values expressed as mean ± SEM (n = 12).*P < 0.05, **P < 0.01 or ***P < 0.001 for the indicated comparison (calculated using a one-way ANOVA test).

As it has been documented that the levels of Aspartate Aminotransferase (AST) and Alkaline Phosphatase (ALP) are modified in a *daf-2* mutant background [37, 38, 39], we aimed to determine any changes in ALP and AST activities in worms fed a HGD. We found that AST activity increased by 20–30% in worms grown at 20, 40 or 80 mM glucose, but showed a decrease of 10% at 100 mM glucose with respect to control (82.7 U/μg protein) (Fig 4A). In the case of ALP, it showed an increase of 20–30% at 20 and 40 mM glucose as compared to control (25 U/μg protein), showing a maximum increase of 45% at 80 mM glucose, and a small increase of 5% at 100 mM glucose (Fig 4B). These results suggest that worms exposed to glucose show increased levels of enzymes associated to an impaired IIS pathway.

### Glucose exposure increased lipid peroxidation

**Fig 5:**
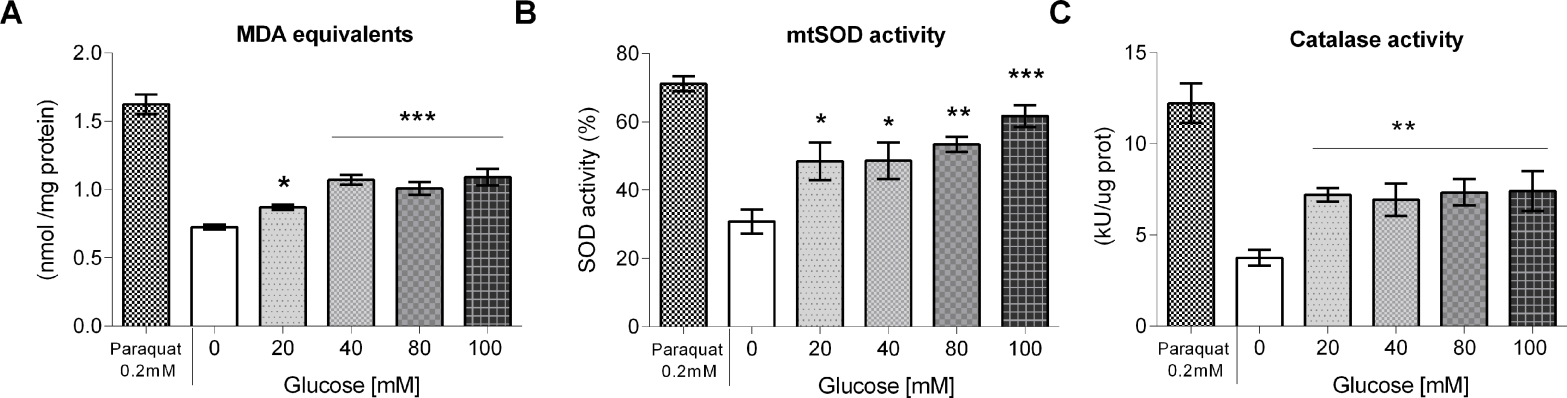
Lipid peroxidation and antioxidant enzymes in glucose-fed worms. Worms were exposed from L1 to L4 larval stage to 20, 40, 80 or 100 mM glucose, then (A) lipid peroxidation, (B) mitochondrial superoxide dismutase (mtSOD), and (C) catalase (CAT) activities were measured. Results are presented as mean ± SEM. In the case of mtSOD, results are presented as %, with controls set to 100%. *P < 0.05, **P < 0.01 or ***P < 0.001 for the indicated comparison (calculated using a one-way ANOVA test).

As HGD are known to generate oxidative stress [3], and oxidative stress is known to cause lipid peroxidation that leads to the formation of malondialdehyde (MDA), we evaluated the MDA equivalents in worms fed a HGD. Fig 5A shows the increase in lipid peroxidation in treated worms with respect to the control (0.72 nmol/mg protein), expressed as percentages. A glucose concentration of 100 mM in the growth media produced the greatest percentage of lipid peroxidation at all the doses assayed (from 19 to 50%). To corroborate that oxidative stress is able to elicit lipid peroxidation, worms were incubated for 1 h in 0.2 mM paraquat, a subtoxic dose that was chosen from a dose-response curve (S1 Fig). We found that control worms treated with paraquat showed an increase in lipid peroxidation as expected (Fig 5A).

### Glucose exposure induced antioxidant enzyme activity, the expression of a *Psod-3*::GFP reporter protein, and increased the glutathione pool

Next, we determined the activities of mtSOD (mitochondrial Superoxide dismutase) and CAT (Catalase), antioxidant enzymes that are normally induced under conditions of oxidative stress. The enzyme activity of mtSOD of larvae treated with 20, 40, 80 or 100 mM glucose was significantly higher (from 57–100%) than that of the control (30.8% of activity). Additionally, CAT activity also increased by 98% with respect to control (3.74 kU/μg protein) at all the glucose concentrations tested (Fig 5C). Paraquat-treated worms showed an increase in both mtSOD and CAT enzyme activities as expected (Fig 5C).

**Fig 6:**
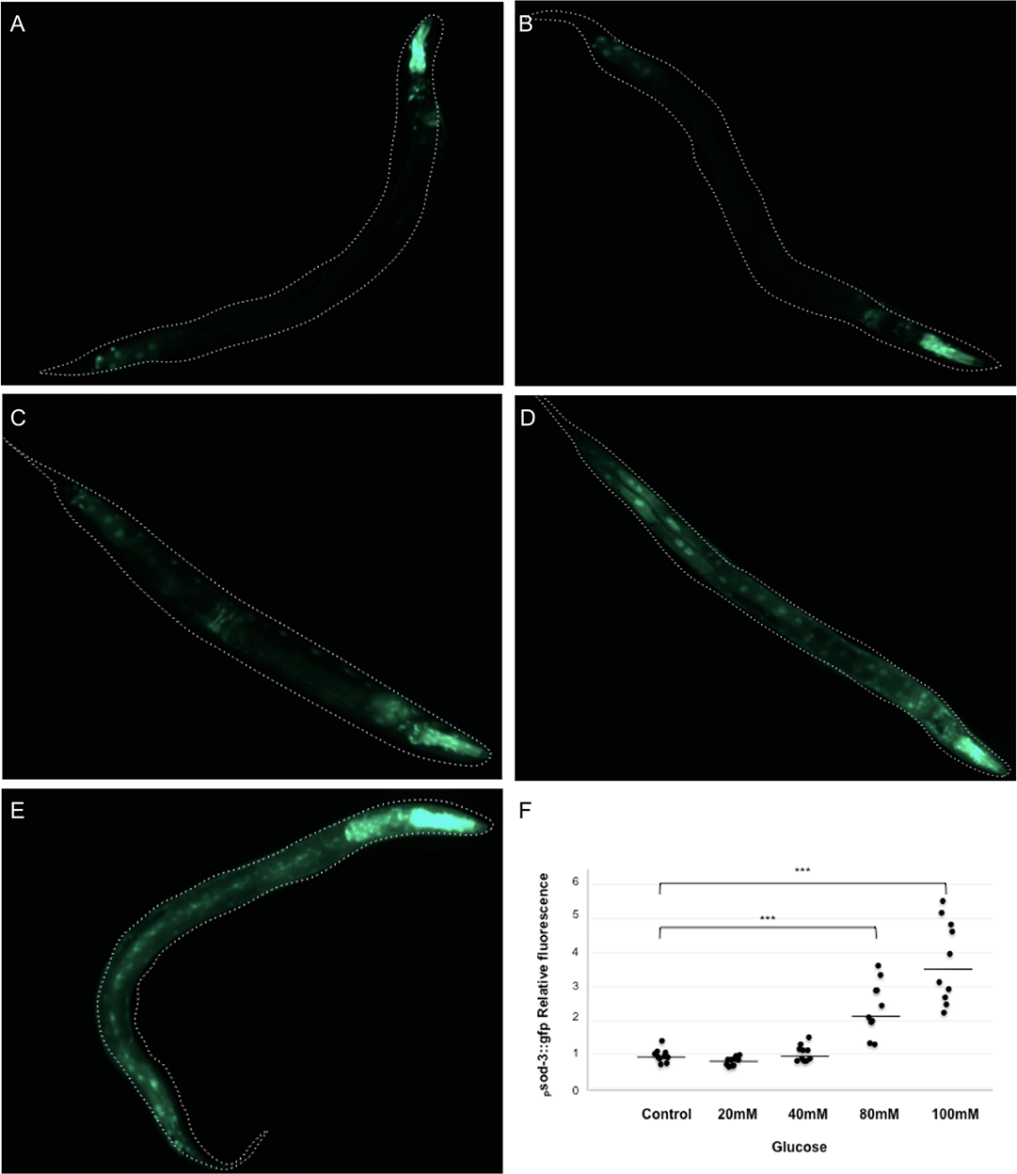
*Psod-3*::*GFP* expression after heat shock is higher when animals were grown in a HGD. (A-E) *Psod-3*::GFP transgen animals were exposed from L1 larvae to L4 larval stage to a diet of 0 (A), 20 (B), 40 (C), 80 (D) or 100 (E) mM glucose. To induce the expression of the reporter, animals were exposed to a heat shock of 31°C for 8 h. After heat shock, animals were mounted and observed for fluorescence. Representative pictures are shown for each case. The data of one of two independent replicates with similar results are shown. (F) Graph shows the relative expression level of each experimental condition. ***P < 0.001 (calculated using a one-way ANOVA test).

As the mtSOD enzyme activity showed an increase in glucose-treated worms, we determined the expression of the *sod-3* gene, as it encodes the mtSOD protein [40], by using a *Psod-3*::GFP fusion protein [41]. For this experiment, we first grew the worms in glucose-additioned media until they reached the L4 larval stage. Then, we induced the expression of *Psod-3*::GFP by heat shock as described in Materials and Methods. We observed that the expression of *Psod-3*::GFP was induced by the heat shock treatment (Fig 6A and 6B). Interestingly, the expression of the *Psod-3*::GFP reporter augmented in a dose-dependent manner as the concentration of glucose in the growth medium, with a maximum at 80 mM glucose, then at 100 mM glucose a small decrease was observed (Fig 6C–6F).

We found that total glutathione, GSH and GSSG levels were significantly increased in worms grown in all of the glucose concentrations tested (Table 1). Interestingly, no changes in the GSH/GSSG ratio were observed under these conditions. As a positive control, worms that were treated with paraquat showed an increase in total glutathione, GSH and GSSG, and a decrease of 20% in the GSH/GSSG ratio, as expected. In conclusion, the observed increase in lipid peroxidation, in mtSOD and CAT enzyme activities, in *sod-3* gene expression, as well as the augmented levels of the glutathione pool, provide evidence that worms fed a HGD show signs of oxidative damage.

**Table 1:** Total glutathione, GSH and GSSG, and GSSG/GSH ratio in glucose-fed worms.

Glucose (mM)	Total glutathione nmol/mg worm	GSH nmol/mg worm	GSSG nmol/mg worm	GSH/GSSG ratio
0	1.77±0.09	1.69±0.09	0.08±0.00	22.33±0.77
20	2.52±0.26^a^ (42)	2.39±0.22^a^(41)	0.14±0.05^b^ (75)	22.64±1.64
40	2.85±0.61^a^ (61)	2.72±0.55^a^ (61)	0.16±0.05^b^ (100)	23.69±3.56
80	3.22±0.24^b^ (82)	3.08±0.25^b^ (82)	0.15±0.01^b^ (87)	22.54±1.29
100	3.19±0.22^b^ (80)	3.09±0.26^b^ (83)	0.12±0.03^b^ (50)	25.91±7.55
0.2mM paraquat	3.22±0.33 (82)	2.81±0.31(66)	0.16±0.02 (100)	17.90±0.32

Effect of increasing concentrations of glucose in the growth medium of *C*. *elegans* on total glutathione, GSH, GSSG, and GSSG/GSH ratio. Data shown in table are mean ± SD; numbers in parentheses represent % of change relative to control (glucose 0 mM)

^a^*P* < 0.05

^b^*P* < 0.01

^c^*P* < 0.001.

### Glucose exposure affected the lifespan of wild-type, mutants or RNAi knock-downs of stress-responsive transcription factors

**Fig 7:**
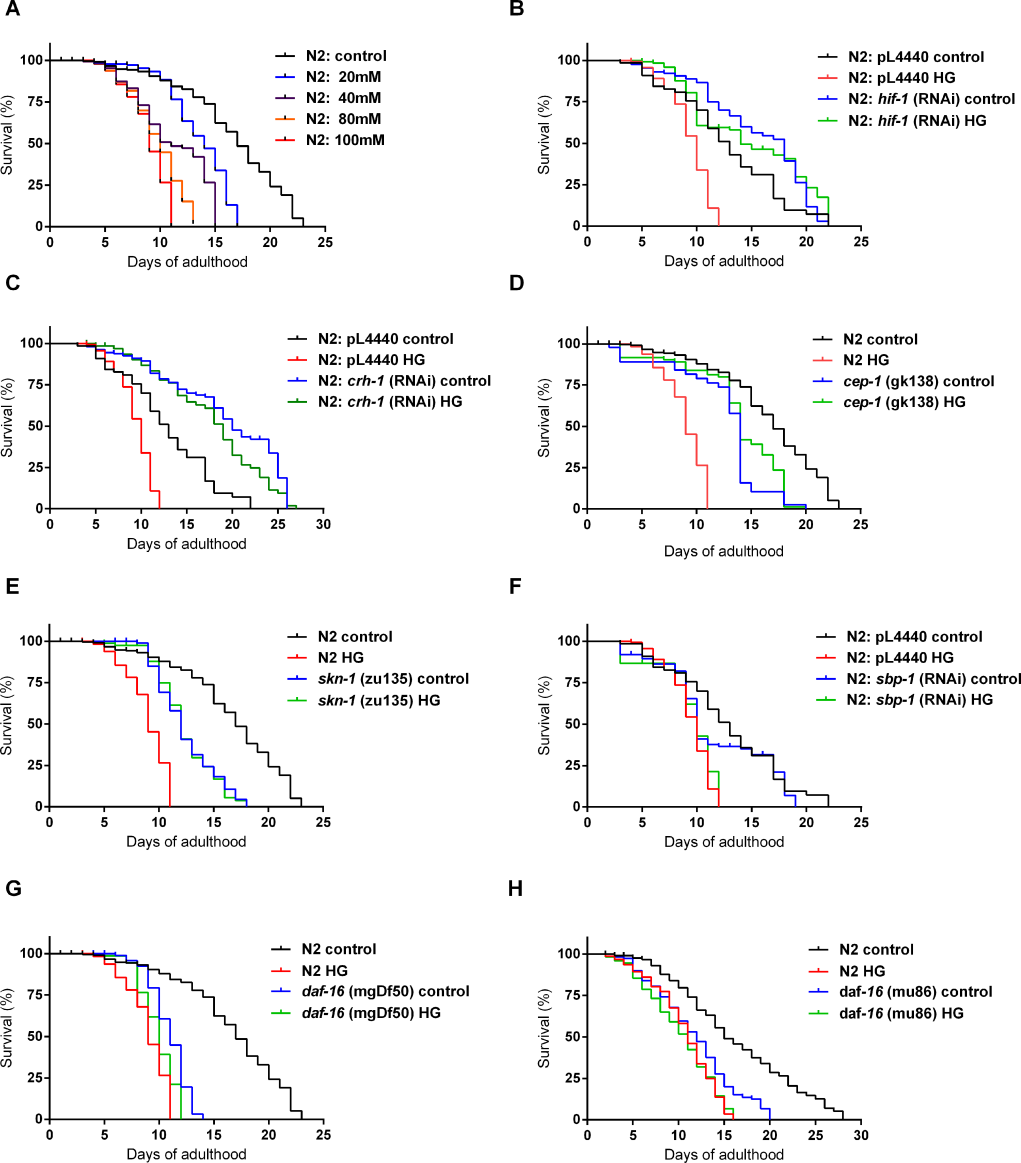
Lifespan of wild-type, mutants or RNAi knock-downs of stress-responsive transcriptional regulators worms upon feeding a HGD. (A) Lifespan curves for adult worms that were exposed from L1 larval stage to the end of their life cycle to 20, 40, 80 or 100 mM glucose (n = 110). (B-H) Lifespan curves for mutants or RNAi knock-downs of adult worms that were exposed from L1 larval stage to the end of their life cycle to 100 mM glucose and compared to controls (n = 110 worms per condition), (B) *hif-1*(RNAi); (C) *crh-1*(RNAi); (D) *cep-1(gk138)*; (E) *skn-1(zu135)*; (F) *sbp-1*(RNAi); (G) *daf-16(mgDf50)*; (H) *daf-16(mu86)*. For RNAi experiments, bacteria containing empty vector pL4400 was used as a control. Differences between groups were calculated using the log-rank test. See Supplemental S2 Table for statistical analysis of lifespan data shown in this figure.

As lifespan is known to be affected by HGD [11,12], we determined how the lifespan of the wild-type strain was altered by growing worms in media additioned with different concentrations of glucose. We found a reduction of 26, 35, 43, and 52% at 20, 40, 80 or 100 mM glucose, respectively, as compared to control (23 days) (Fig 7A and S2 Table).

As a way to find out if transcription factors (TFs) known to be key regulators of carbohydrate and lipid metabolism, oxidative stress and longevity, such as HIF-1/HIF1α, CRH-1/CREB, CEP-1/p53, SKN-1/NRF2, SBP-1/SREBP, and DAF-16/FOXO, were involved in the glucose-dependent reduction in lifespan observed in the wild-type strain, we assessed the longevity of mutants or RNAi knock-downs of the previously mentioned TFs in worms grown at 100 mM glucose (Fig 7B–7H and S2 Table). We found that the lifespan for *hif-1*(RNAi) and *crh-1*(RNAi) grown in glucose was longer than the lifespan of the wild-type strain grown in the absence of glucose (Fig 7B and 7C, respectively; S2 Table), while the lifespan of *cep-1(gk138)* and *skn-1(zu135)* glucose-fed worms was similar to that shown by the wild-type strain grown in the absence of glucose (Fig 7D and Fig 7E, respectively; S2 Table). The lifespan of *sbp-1*(RNAi) and two different mutants in *daf-16*, *daf-16(mgDf50)* and *daf-16(mu86)* grown in glucose-rich media was similar to the lifespan of the wild-type strain in the presence of glucose (Fig 7F, 7G and 7H, respectively; S2 Table). These findings support the hypothesis that the shortening of lifespan is mediated by convergent signaling pathways.

### Glucose exposure affected the accumulation of several mRNAs that code for stress-responsive transcription factors

**Fig 8:**
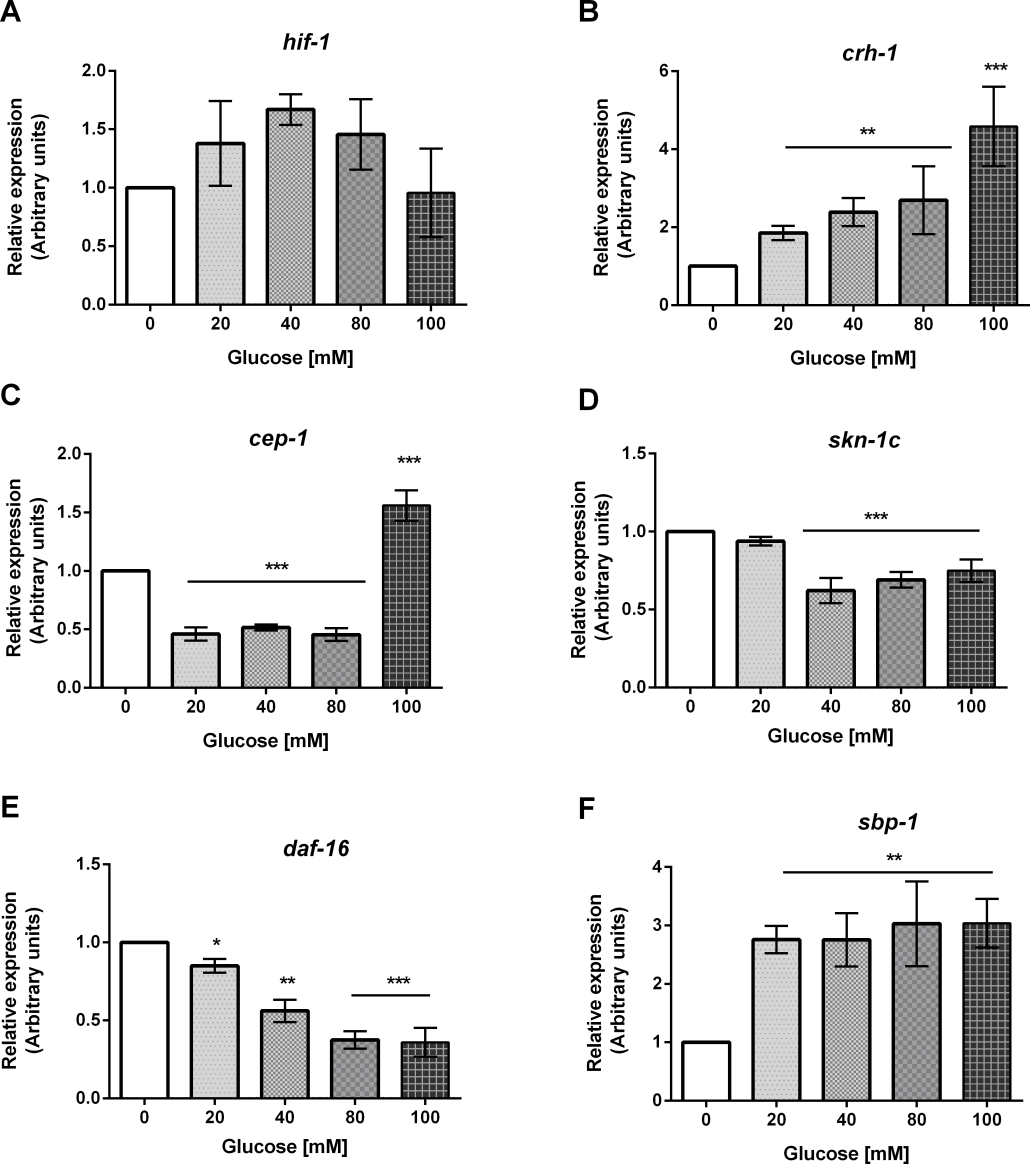
mRNA abundance of stress-responsive transcription factors of worms grown at different concentrations of glucose. Worms were exposed from L1 to L4 larval stage to 20, 40, 80 or 100 mM glucose. Panels show quantitative RT-qPCR analysis of: (A) *hif-1*; (B) *crh-1*; (C) *cep-1*; (D) *skn-1c*; (E) *daf-16*; (F) *sbp-1* mRNA level in wild-type worms grown at the specified glucose concentration. Values expressed as median ± IQR (n = 6).*P < 0.05, **P < 0.01 or ***P < 0.001 for the indicated comparison (calculated using the Kruskal-Wallis test).

Taking into consideration the longevity results of the mutants and RNAi knock-downs of the stress-responsive TFs studied, we aimed to explore if some of the observed changes in glucose-fed worms could be related to changes in the expression of *skn-1* (isoform c), *hif-1*, *sbp-1*, *crh-1*, *cep-1*, and *daf-16* (isoforms a, b, d, and f), which are involved in the regulation of metabolism, longevity, lipid accumulation, and oxidative stress (Fig 8 and S2 Table). We found that mRNA accumulation showed no change for *hif-1*/HIF1α (Fig 8A), while it was increased for *crh-1*/CREB (Fig 8B), as glucose concentration in the growth media was increased. For *cep-1*/p53, mRNA accumulation was around 50% less than in the control at 20, 40 or 80 mM glucose, but was 50% more than the control at 100 mM glucose (Fig 8C). For *skn-1*/NRF2 and *daf-16*/FOXO, mRNA accumulation was decreased in glucose-fed worms in a dose-dependent manner (Fig 8D and 8E, respectively). In the case of *sbp-1*/SREBP, mRNA accumulation was 176% or 200% higher than the control if worms are grown at 20–40 mM glucose, or 80–100 mM glucose, respectively (Fig 8F). Taken together, except for *hif-1* mRNA, mRNA accumulation of the stress-responsive transcription factors analyzed was modulated by glucose treatment: it was increased for *crh-1* and *sbp-1*, and was decreased for *cep-1*, *skn-1*, and *daf-16*, probably as part of a mechanism in which several signaling pathways participate to regain homeostasis in worms fed a HGD.

## Discussion

In our study, worms were grown from L1 to L4 larval stages in glucose-supplemented growth media (20, 40, 80 or 100 mM). Glucose-fed worms showed biochemical and physiological alterations as compared to worms grown without added glucose. First, we confirmed that glucose-fed worms accumulated glucose into their bodies in a concentration-dependent way, from 20 to 80 mM glucose, showing a ten-fold increase as compared to control. Interestingly, glucose accumulation was the same in worms grown in 80 or 100 mM glucose. We can exclude differences in glucose uptake at 80 and 100 mM glucose as a possible explanation, as glucose uptake was linearly dependent on glucose concentration in the growth media. To our knowledge, this is the first time that glucose uptake has been determined in experiments with worms fed a HGD. Also, we observed that glucose uptake increased 14-fold if worms were treated with 80 mU of insulin for 2 h, suggesting that glucose uptake is regulated by the IIS pathway. This result is consistent with previous reports in which the expression of FGT-1, the primary glucose transporter of *C*. *elegans*, was shown to be under the control of the insulin/IGF-like signaling pathway [42].

Additionally, as glucose is a known precursor of triglycerides, we determined the triglyceride content in glucose-fed worms. We found that the triglyceride content increased in a concentration-dependent manner from 20 to 80 mM glucose. Like glucose accumulation, the triglyceride content was similar at 80 and 100 mM glucose, maybe as a consequence of the fact that glucose accumulation is similar at 80 or 100 mM glucose. Our results suggest that high levels of accumulation of glucose promote lipogenesis by increasing the triglyceride pool, similar to what has been found in mammals [43].

As food limitation is known to result in a reduced body size [35], we determined if a HGD produced alterations in body size. We found that worms fed a HGD were larger and thicker compared to worms grown in the absence of glucose (see Fig 2). This is in line with previous reports that show the influence of nutrient content on body size [44].

Glucose exposure also had effects on the amount of eggs laid. When we exposed worms of the P0 generation to glucose, we did not find any change in the number of eggs laid. However, when we exposed to glucose worms at the P0 generation and then raised the F1 and F2 generations in glucose-supplemented growth medium, the amount of eggs laid in the F1 and F2 generations was decreased, suggesting that changes produced by glucose exposure can be inherited to other generations. A similar situation as has been reported recently, in which worms grown in 2% glucose (approximately 111 mM) in the P0 generation showed adverse effects in fecundity [45]. Taking into account that glucose-fed worms showed an increased triglyceride content, it is possible that the reduced number of eggs laid is a reflection of the increased synthesis and accumulation of fat, as it has been shown in *C*. *elegans* that fatty acids play important roles in fertility [46] and sperm motility [47].

We then attempted to explain the reduction in the amount of eggs laid in glucose-fed worms, so we documented the presence of egg-laying defects in the form of egg retention with internal hatching or “bagging”, in which embryos hatch inside the worms, killing them. We observed a small increase in “bagging” in glucose 40, 80 or 100 mM. This phenotype is similar to what has been observed in worms fed with glucose 100 mM, when alterations in egg-laying were associated with an impairment in the serotoninergic signaling pathway that control vulval muscles [48].

As the effects of added glucose on glucose content, brood size, lifespan, and oxidative stress have been studied in different labs under diverse experimental conditions, we provide here a table that compares our results with the results obtained in other studies (Table 2).

**Table 2:** Comparison of our findings with existing studies on glucose content, brood size, lifespan and oxidative stress on worms fed different concentrations of glucose.

**GLUCOSE CONTENT**				
***Glucose concentration in growth media***	***Treatment***	***Results***	***Comments***	***References***
10, 20, 30, 40, or 50 mM	Worms were cultured for 5 days on agar plates containing various concentrations of glucose (n = 100)	Total body glucose concentrations in the range of 7 to 15 mM were obtained.	High glucose conditions resulted in total body glucose concentrations similar to the ones observed in diabeteic patients under poor glucose control.	[1]
2% (111 mM)	Adults (1 day)	Total body glucose increased by 50%.	Glucose content was evaluated in adult worms. High glucose diet increased total body glucose.	[3]
2% (111 mM)	Worms were grown on glucose from hatching to young adults.	Internal glucose levels were increased fourfold (from 132 to 737 nmol glucose/mg protein).	High glucose diet increases internal glucose level.	[5]
100 or 200 mM	Worms were fed with glucose from the L1 stage, then L4 worms were loaded into a microdevice (n>30)	Total body glucose concentrations of 6.7 or 12 mM when worms fed glucose 100 or 200 mM, respectively.	Glucose feeding produced total body glucose concentrations similar to the ones observed in poorly controlled diabetic patients.	[9]
Not specified.	Worms were cultured under high glucose conditions.	Total body glucose concentrations of 13 mM.	Glucose feeding produced total body glucose concentrations similar to the ones observed in poorly controlled diabetic patients.	[7]
**20, 40, 80, or 100 mM**	**Worms were fed with glucose from the L1 stage until the L4 stage.**	**Glucose content increased 2-, 3-, 14- or 11-fold as compared to control (16.63 mg/dL = 0.9 mM)**	**Internal glucose increased as glucose augmented in growth media, reaching a plateau at 80 mM.**	**This work.**
**BROOD SIZE**				
100 mM	L4 larvae.	Brood size was similar to control.	Reductions in egg-laying rate and number of eggs in utero.	[4]
0.1, 1, 2, 4, or 10% (5.5, 55, 111, 222, or 555 mM)	L4 larvae (n = 10)	Brood size was reduced by 34%, 52%, or 74% on glucose 5.5 mM, 55–111 mM, or 222–555 mM	Worms were grown on NGM plus each glucose condition and 2 next days progeny was scored for each experiment. Replicates are not declared.	[5]
2% (111 mM)	L4 larvae (P0) exposed to glucose, then F1, F2 and F3 generations were grown in the absence of glucose (n = 10)	Brood size was reduced in glucose-fed worms in the P0, F1 and F2 generations, although only the P0 generation was exposed to glucose.	Glucose induced a transgenerational reduction in brood size from a single exposure to glucose at the P0 generation.	[6]
2% (0.111 mM)	L4 larvae (n = 36)	Brood size was reduced by 26%.	They used FUDR to pre-fertile young adults to prevent their progeny from developing.	[3]
50, 125, 250, 333, or 550 mM	Worms were grown from L1 to L4 stage in high-glucose diets.	Brood size was not reduced in worms grown in <250 mM glucose; a reduction in fertility by 25% or 60% was observed in glucose concentrations of 333mM or 500 mM glucose, respectively.	O-linked-N-acetylglucosamine cycling and insulin signaling are required for an adequate response to high glucose diets.	[2]
**20, 40, 80, or 100 mM**	**Worms were fed with glucose from the L1 stage until the L4 stage for each generation (n = 30).**	**Brood size was not reduced in the P0 generation, but showed a decrease in the F1 generaton and a further decrease in the F2 generation.**	**Brood size was reduced in part as an effect of an increase in “bagging”.**	**This work.**
**LIFESPAN**				
40 mM	Adults (day 1) until death (n = 100).	Mean lifespan and maximum lifespan were reduced by 11% and 10%, respectively.	FudR was added to media to to prevent progeny production respectively. Growth in a high glucose diet reduced lifespan.	[1]
Not specified.	Adults (1 day) until death (n = 50)	Mean and maximum lifespan was reduced by 13% or 14%, respectively.	FudR were added to media to prevent eggs from hatching. Human insulin treatment increased shortened mean and maximum lifespan in worms fed high glucose diets.	[7]
0.1, 1, 2, 4, or 10% (5.5, 55, 111, 222, or 555 mM)	Adults (day 1) until death (n = 20).	No negative effects were observed in lifespan of worms grown in up to111 mM glucose; however, significant reduction was observed in worms fed with 222 or 555 mM of glucose.	FudR were added to media to prevent hatching.	[5]
2% (111 mM)	Adults (1 day) until death	Lifespan reduction of 20%.	Lifespan reduction only observed when glucose was administered during adulthood, not when feeding during development.	[3]
4% (222 mM)	Worms were grown in glucose to L4 and then transferred to glucose-free media (n = 20)	Mean and maximum lifespan was reduced by 17% and 22%, respectively.	FudR was added to the media. Glucose exposure reduced the lifespan in the P0 generation, but this trait was not passed to F1 or F2 generations.	[6]
1, 10 or 20 mg/L (0.005, 0.055, or 0.111 mM)	Adults were fed with glucose from day 1 until death (n = 100)	Lifespan reduction of 23% or 49.5% at glucose 0.055 or 0.111 mM, respectively.	Dose-dependent reduction in lifespan was associated with ectopic apoptosis in the body.	[8]
100 or 200 mM	Worms were fed with glucose from the L1 stage, then L4 worms were loaded into microdevice until death (n>30)	Lifespan reduction of 29% or 31% at glucose 100 or 200 mM, respectively.	Reduction in lifespan in high-glucose conditions was associated with increased expression of oxidative stress response proteins and fat metabolism genes.	[9]
**20, 40, 80, or 100 mM**	**Worms were fed with glucose from the L1 stage until the L4 stage for each generation (n = 110).**	**Lifespan was reduced by 26%, 35%, 43%, or 52% at 20, 40, 80 or 100 mM glucose, respectively**	**Lifespan was reduced in all glucose conditions in a concentration-dependent manner.**	**This work.**
**OXIDATIVE STRESS**				
Not specified	Adults (1 day)	*sod-3* mRNA and SOD activity were reduced by 69% and 28%, respectively; ROS was augmented by 71%.	Quantification of ROS was performed with hydroethidine staining. High glucose diets increase ROS.	[7]
100 or 200 mM	Worms were fed with glucose from the L1 stage, then L4 worms were loaded into microdevice (n>30)	Dose-dependent increased expression of *gst-4*::GFP	Growth in a high glucose diet produced an increase in oxidative stress.	[9]
2% (111 mM)	Adults	Glucose did not induce a generalized oxidative stress.	Quantification of ROS was performed with dihydrofluorescein staining. Glucose induced resistance against oxidative stress on progeny from a unique exposure event on the P0 generation.	[6]
40 mM	Adults cultured for 15 days under high glucose conditions.	ROS was increased by 95%.	Quantification of ROS was performed with dihydroethidium staining. Growth in a high glucose diet increased ROS levels.	[1]
**20, 40, 80, or 100 mM**	**Worms were fed with glucose from the L1 stage until the L4 stage.**	**Glucose exposure augmented lipid peroxidation, as well as CAT and mtSOD enzymatic activities, total GSH, and *Psod-3*::GFP expression.**	**Oxidative stress markers were increased in all conditions.**	**This work.**

### HGD induced alterations associated with an impaired insulin/insulin-like growth factor (IGF) signaling (IIS) pathway

As it has been reported that AST and ALP activities are dramatically changed in IIS pathway mutants [37, 38, 39], we determined if glucose exposure caused alterations in these enzyme activities. We found that AST and ALP activities were increased in worms grown in glucose, a known modulator of the IIS pathway. To our knowledge, this is the first report that shows that AST and ALP enzyme activities are induced in glucose-fed worms. The increase in activity of AST and ALP in glucose fed-worms could be interpreted as a sign of aging, as a similar increase is observed in aged worms [38, 39], and could indicate that glucose exposure is accelerating aging in worms, as has been hypothesized previously [11, 49]. More experiments should be performed to get a firmer argument on this topic.

### HGD induced oxidative stress

Additionally, it has been documented that a HGD can increase the generation of ROS [50], but these studies have not characterized in detail the presence of oxidative stress nor the induction of components of the antioxidant system. We found evidence that glucose-fed worms are experincing oxidative stress based on the following considerations: first, we showed that glucose-fed worms are facing some sort of molecular damage, as lipid peroxidation was increased. Such an increase in lipid peroxidation has been observed in worm´s homogenates exposed to copper, where it was used for quantification of oxidative damage in the worm [51]. Second, glucose-fed worms showed increased activities of mtSOD and CAT, enzymes that have an important participation in the antioxidant system. Furthermore, we found that the expression of *sod-3*, the gene that codes for mtSOD, was induced in a dose-dependent manner in worms fed a HGD. Although our results are contrary to those reported by Mendler et al. [23], who observed no changes in SOD activity in glucose-fed worms, the difference could be attributable to the fact that we measured mitochondrial SOD activity, while Mendler et al. [23] determined total SOD activity. Third, previous studies in *C*. *elegans* have shown that, similar to mammals, glutathione metabolism is modulated by oxidative stress [52, 53]. In our study, we found that total glutathione, GSH and GSSG levels were increased in worms fed a HGD, suggesting that glucose-fed worms are experiencing oxidative stress. Interestingly, the ratio of GSH/GSSG remained unchanged under all the conditions analyzed, indicating that worms exposed up to 100 mM glucose were still able to induce the glutathione system as a defense mechanism against oxidative stress. Taken together, these data suggest that a HGD induced oxidative stress, and that different antioxidant systems are activated to mount a response.

### Convergent pathways modulate the lifespan of worms fed a HGD

We also observed that glucose-fed worms showed a decreased lifespan. Most of the lifespan assays in *C*. *elegans* have been routinely performed using the compound 5-fluoro-2´-deoxyuridine (FUdR) to prevent eggs from hatching, but recent reports have shown that FUdR treatment alter stress responses and metabolism, causing misleading results on lifespan determination [54–56]. Here we assayed lifespan without using FUdR and found that wild-type glucose-fed worms showed a pronounced decrease in a dose-dependent manner. Maximal reduction was 52% at 100 mM glucose. A similar decrease in lifespan has been previously described [57].

Next, we aimed to document the participation of stress-responsive transcription factors such as SKN-1/NRF2, HIF-1/HIF1α, SBP-1/SREBP, CRH-1/CREB, CEP-1/p53, and DAF-16/FOXO, in the decrease of lifespan induced by glucose 100 mM. To our knowledge, this is the first time that the effect of glucose on mutants or RNAi knock-downs of *hif-1*, *crh-1*, *cep-1* or *skn-1* genes is reported. It is interesting to note that, some mutants or RNAi strains when grown in glucose, have a longer (*hif-1*(RNAi) and *crh-1*(RNAi)) or similar lifespan (*cep-1(gk138)* and *skn-1(zu135)*) than the wild-type strain grown in the absence of glucose. We interpret these results as a sign that inactivation of *hif-1*, *crh-1*, *cep-1* and *skn-1* have a protective effect on the lifespan of glucose-fed worms. Our results suggest that the pharmacological inactivation of HIF-1α, CREB, p53 or NRF2 could be beneficial for the treatment of obesity and type 2 diabetes in humans.

In the case of *sbp-1*(RNAi) and *daf-16(mgDf50)*, their lifespan when grown in glucose was similar to the lifespan of the wild-type strain grown in glucose, so in this case, glucose did not further decrease the lifespan of *sbp-1*(RNAi) and *daf-16(mgDf50)* when grown in the absence of glucose. The lifespan of *sbp-1*(RNAi) grown in the presence of glucose was similar to that reported by Lee et al. [41]. For the *daf-16(mgDf50)* mutant, the observed result is similar to what has been reported elsewhere [58, 59]. We also observed that when grown at glucose 100 mM, the *daf-16(mgDf50)* mutant showed a slight decrease in both the mean and maximum lifespan compared to the condition without added glucose. This is in contrast to what has been reported, as Lee et al. did not found a further shortening of the lifespan of a *daf-16* mutant by growing the worms in a HGD [11]. The difference observed could be related to the fact that Lee et al. used a different mutant strain, e.g. *daf-16(mu86)* [11]. In order to elucidate this matter, we also assesed the lifespan of the *daf-16(mu86)* mutant and observed that, in our hands, both mean and maximum lifespan were reduced when this mutant strain was grown in 100 mM glucose, in a similar percentage as the decrease that we found in the *daf-16(mgDf50)* mutant strain. Taken together, the above results suggest that several convergent pathways participate in the modulation of lifespan in worms fed a HGD.

### mRNA accumulation of several stress-responsive transcription factors was modulated by a HGD

We then wanted to gain insight into the changes in the mRNA accumulation of the stress-responsive transcription factors analyzed in the lifespan studies. For most of them, this is the first time that the mRNA accumulation has been studied, as some of the previous expression studies have relied mainly on GFP-reporter gene expression assays. We observed no change in the case of *hif-1* mRNA. Maybe this result could be explainded by the fact that the main way to regulate the activity of HIF-1 is through phosphorylation and ubiquitin-dependent degradation [60].

For the other transcription factors, we found that the mRNA abundance was increased for *crh-1* and *sbp-1*, while it was decreased for *skn-1*, *daf-16* and *cep-1* when worms were fed a HGD. The result that *daf-16* and *skn-1* mRNA levels are downregulated is interesting as a recent paper shows that DAF-16/FOXO can directly activate the expression of *skn-1* [61]. Furthermore, it has been reported that 10-day adult worms are unable to mount an adaptive response to oxidative stress, as old worms showed a reduced expression of DAF-16 and SKN-1 regulated genes [62]. This is also in line with the idea that worms fed a HGD show something reminiscent of an accelerated aging.

Additionally, we observed that *crh-1* expression augmented in a linear fashion with the concentration of glucose in the growth medium. It has been documented that CRH-1 autoregulates its own expression, with the help of the coactivator CRTC-1/CRTCs [63]. In our experiments, we also observed that the mRNA abundance of *cep-1* was downregulated in glucose-fed worms. Interestingly, this downregulation could be explained as it has been reported that CRTC-1/CRTCs acts also as a negative regulator of *cep-1* expression [64]. Also, we observed an increase in mRNA abundance of *sbp-1* in worms exposed to glucose. This finding is consistent with an observed increase in the abundance of an SBP-1::GFP fusion protein in glucose-fed worms [49]. Clearly, more profound studies are needed in order to link the changes in mRNA abundance of the stress-responsive transcription factors analyzed with some of the many effects that dietary glucose has on several processes, such as membrane fluidity [65], glycogen accumulation [66], accumulation of unsaturated fatty acids and dihydroxyacetone phosphate [49], and accumulation of advanced glycation end products (AGEs) [12].

## Conclusions

We have shown that glucose-fed worms show altered growth, development, aging and reproduction, when grown in increasing concentrations of glucose from L1 to L4 stages. Additionally, we present evidence that glucose-fed worms show signs of accelerated aging, a rise in lipoperoxidation, an increase in the activity of some enzymes of the antioxidant system (mtSOD and CAT), and an increase in the glutathione pool content, and an induced expression of *sod-3*, which suggests that glucose-fed worms are experiencing oxidative stress. Furthermore, we show evidence of the participation of several stress-responsive transcriptional regulators in lifespan reduction in glucose-fed worms. Our results suggest that the response to glucose is orchestrated by a regulatory network in which several signaling pathways participate, in addition to the well known IIS pathway. As the stress-responsive transcription factors analyzed are involved in pathways that are conserved from worms to humans, this knowledge could be used in the design of therapies for obesity and type 2 diabetes in humans.

## Supporting information

S1 Fig Effect of different doses of paraquat on lipid peroxidation levels. To induce oxidative stress, L4 worms grown in NGM were incubated for 1 hour at 20°C in PBS 1X containing 0.05, 0.1, 0.2, 0.5, 1.0, or 2.0 mM paraquat. After this treatment, worms were washed 3 times with PBS 1X to remove any traces of paraquat, and then MDA content was measured (see Material and Methods). We chose 0.2 mM paraquat to induce oxidative stress, as in this concentration we observed the maximum level of MDA. Experiments were performed six times. Values are expressed as mean ± SEM. Significant differences with respect to control group are marked as follows: *P < 0.05, **P < 0.01, or ns = non-significant, for the indicated comparison. Data was analyzed by one-way ANOVA test.
(TIF)

S1 Table List of primers. Pairs of primers used in qRT-PCR analysis.
(DOCX)

S2 Table Survival data and statistics for lifespan experiments. The effects of glucose-supplemented diets on the lifespan of wild-type, mutant or RNAi-treated animals. Lifespan was evaluated in triplicates for each condition and strain (see Material and Methods). For each analysis, 110 worms were used. Statistical evaluation of lifespan assays were made using the Log-rank (Mantel-Cox) test, differences between groups were evaluated with the Bonferroni *post hoc* test. *P*<0.05 was regarded as statistically significant.
(PDF)
