# Supplementary material for: Caenorhabditis elegans respond to high-glucose diets through a network of stress-responsive transcription factors
Source: PLoS One. 2018 Jul 10;13(7):e0199888. doi: 10.1371/journal.pone.0199888 (PMC6039004; doi:10.1371/journal.pone.0199888)
Supplement: S1 Table — Pairs of primers used in qRT-PCR analysis. (DOCX) [file pone.0199888.s002.docx]

**S1 Table. Primers used for RT-qPCR.**

| Gene | Sequence | Concentration (nM) | Tm (°C) |
| --- | --- | --- | --- |
| *Y45F10D.4* | *Fw* gcgaaaacactcctgcac  *Rev* tttcgcgggttctcgtagtg | 66 | 60 |
| *sbp-1* | *Fw* CTT CTC AAT GCG ATT TTC CC  *Rev*   TCC AGA TAA TTG TTG GGT GG | 66 | 55 |
| *crh-1* | Fw ATG GAG TCA CTG GTT TTC AAT  Rev TTG TGG TCC TCC TGG AAA TA | 66 | 55 |
| *hif-1* | Fw CGG AAA AGA AAC ATG GAA CG  Rev CGA TTC TTT AGA CCG ACG AT | 66 | 55 |
| *cep-1* | Fw CCC AAG TTT GAG CGC AGA ATG  Rev CCG ATG TCC TGC GAA TCG GG | 66 | 60 |
| *skn-1c* | Fw AAG GGC ACA CGA CAA GTG G  Rev AGC ATT CTC TTC GGC AGT GAG | 66 | 60 |
| *daf-16* | Fw AAA GAG CTC GTG GTG GGT TA  Rev TTC GAG TTG AGC CTT TGT AGT CG | 66 | 63 |
