# Supplementary material for: Caenorhabditis elegans respond to high-glucose diets through a network of stress-responsive transcription factors
Source: PLoS One. 2018 Jul 10;13(7):e0199888. doi: 10.1371/journal.pone.0199888 (PMC6039004; doi:10.1371/journal.pone.0199888)
Supplement: S2 Table — The effects of glucose-supplemented diets on the lifespan of wild-type, mutant or RNAi-treated animals. Lifespan was evaluated in triplicates for each condition and strain (see Material and Methods). For each analysis, 110 worms were used. Statistical evaluation of lifespan assays were made using the Log-rank (Mantel-Cox) test, differences between groups were evaluated with the Bonferroni post hoc test. P<0.05 was regarded as statistically significant. (PDF) [file pone.0199888.s003.pdf]

S2 Table. Survival data and statistics for lifespan experiments. The effects of glucose-supplemented diets on the lifespan of wild-type, mutant or RNAi-treated animals.

Treatment	Strain (n)	Mean lifespan (days)	Changes in mean lifespan (%) [Δ]	Max. lifespan (days)	Changes in max lifespan (%) [Δ]	χ^2	*P* value	Figure in text
Control	N2 Bristol (110)	14.48	-	23	-	-	-	Figure 7A
	N2 Bristol (110)	16.18	-	26	-	-	-	Figure 7A
	N2 Bristol (110)	17.21	-	25	-	-	-	Figure 7A
20 mM glucose	N2 Bristol (110)	14.11	-2.56 [a]	17	-26.09 [a]	0.23 [a]	0.628 (ns)	Figure 7A
	N2 Bristol (110)	16.21	+0.19 [a]	19	-26.92 [a]	1.33 [a]	0.479 (ns)	Figure 7A
	N2 Bristol (110)	16.40	-4.71 [a]	20	-20.00 [a]	1.19 [a]	0.316 (ns)	Figure 7A
40 mM glucose	N2 Bristol (110)	10.03	-30.73 [a]	16	-30.43 [a]	48.72 [a]	<0.0001	Figure 7A
	N2 Bristol (110)	11.22	-30.66 [a]	15	-42.31 [a]	52.65 [a]	<0.0001	Figure 7A
	N2 Bristol (110)	13.23	-23.13 [a]	15	-40.00 [a]	50.37 [a]	<0.0001	Figure 7A
80 mM glucose	N2 Bristol (110)	9.72	-32.87 [a]	13	-43.48 [a]	57.72 [a]	<0.0001	Figure 7A
	N2 Bristol (110)	12.43	-23.18 [a]	13	-50.00 [a]	65.96 [a]	<0.0001	Figure 7A
	N2 Bristol (110)	11.80	-31.44 [a]	16	-36.00 [a]	47.60 [a]	<0.0001	Figure 7A
100 mM glucose	N2 Bristol (110)	9.25	-36.12 [a]	11	-52.17 [a]	71.63 [a]	<0.0001	Figure 7A
	N2 Bristol (110)	10.02	-38.07 [a]	13	-50.00 [a]	80.37 [a]	<0.0001	Figure 7A
	N2 Bristol (110)	11.49	-33.24 [a]	15	-40.00 [a]	60.47 [a]	<0.0001	Figure 7A
Control	N2 Bristol (100)	14.43	-	23	-	-	-	Figures 7D, 7E, and 7G,
	N2 Bristol (120)	16.24	-	26	-	-	-	Figures 7D, 7E, and 7G,
	N2 Bristol (120)	17.21	-	27	-	-	-	Figures 7D, 7E, and 7G,
100 mM glucose	N2 Bristol (110)	9.79	-32.15 [a]	11	-52.17 [a]	39.14 [a]	<0.0001	Figures 7D, 7E, and 7G,
	N2 Bristol (120)	10.70	-34.11 [a]	16	-38.46 [a]	86.79 [a]	<0.0001	Figures 7D, 7E, and 7G,
	N2 Bristol (120)	10.02	-38.30 [a]	13	-51.85 [a]	56.69 [a]	<0.0001	Figures 7D, 7E, and 7G,
Control	*daf-16* (mgDf50) (110)	10.95	-24.11 [a]	14	-39.13 [a]	46.09 [a]	<0.0001	Figure 7G
	daf-16 (mgDf50) (110)	11.03	-32.08 [a]	16	-38.46 [a]	44.28 [a]	<0.0001	Figure 7G
	daf-16 (mgDf50) (110)	11.45	-33.47 [a]	16	-40.74 [a]	53.27 [a]	<0.0001	Figure 7G

100 mM glucose	*daf-16* (mgDf50) (110)	9.91	-31.32 [a]	12	-47.83 [a]	26.69[a]<br>11.75[b]	<0.0001<br>0.0018	Figure 7G
	daf-16 (mgDf50) (110)	8.62	-46.92 [a]	13	-50.00 [a]	30.18 [a]<br>13.27 [b]	<0.0001<br>0.0029	Figure 7G
	daf-16 (mgDf50) (110)	10.2	-40.73 [a]	11	-59.26 [a]	37.64 [a]<br>16.50 [b]	<0.0001<br>0.0003	Figure 7G
Control	*daf-16* (mu86) (120)	11.85	-17.88 [a]	20	-13.04 [a]	51.35 [a]	<0.0001	Figure 7H
	daf-16 (mu86) (120)	11.26	-30.67 [a]	20	-23.08 [a]	54.63 [a]	<0.0001	Figure 7H
100 mM glucose	*daf-16* (mu86) (120)	10.23	-29.11 [a]	16	-30.43 [a]	94.61 [a]<br>13.04 [b]	<0.0001<br>0.0003	Figure 7H
	daf-16 (mu86) (120)	9.19	-43.41 [a]	15	-42.30 [a]	74.80 [a]<br>17.26 [b]	<0.0001<br>0.0005	Figure 7H
Control	*skn-1* (zu135) (110)	12.41	-13.99 [a]	18	-21.74 [a]	18.59 [a]	<0.0001	Figure 7E
	skn-1 (zu135) (110)	12.23	-24.69 [a]	18	-30.77 [a]	19.78 [a]	<0.0001	Figure 7E
	skn-1 (zu135) (110)	12.16	+18.93 [a]	19	-20.83 [a]	16.54 [a]	0.0002	Figure 7E
100 mM glucose	*skn-1* (zu135) (110)	12.35	-14.41 [a]	18	-21.74 [a]	15.99[a]<br>0.01[b]	0.0002<br>0.904 (ns)	Figure 7E
	skn-1 (zu135) (110)	12.40	-23.65 [a]	18	-30.77 [a]	17.21 [a]<br>0.9 [b]	0.0001<br>0.183(ns)	Figure 7E<br>Figure 7E
	skn-1 (zu135) (110)	11.70	-27.96 [a]	17	-29.16 [a]	16.47 [a]<br>0.18 [b]	0.0002<br>0.234(ns)	Figure 7E<br>Figure 7E
Control	*cep-1* (gk138) (110)	13.79	-4.44 [a]	20	-13.04 [a]	13.26[a]	0.0008	Figure 7D
	cep-1 (gk138) (110)	14.02	-13.67 [a]	22	-15.38 [a]	16.47 [a]	0.0003	Figure 7D
	cep-1 (gk138) (110)	14.22	-17.37 [a]	21	-22.22 [a]	18.97 [a]	0.0001	Figure 7D
100 mM glucose	*cep-1* (gk138) (110)	12.49	-13.44 [a]	20	-13.04 [a]	3.54[a]<br>4.97[b]	0.179 (ns)<br>0.078 (ns)	Figure 7D
	cep-1 (gk138) (110)	13.06	-19.58 [a]	19	-26.92 [a]	6.48 [a]<br>2.68 [b]	0.0053(ns)<br>0.086(ns)	Figure 7D<br>Figure 7D
	cep-1 (gk138) (110)	12.7	-26.21 [a]	21	-22.22 [a]	4.16 [a]<br>1.27 [b]	0.093 (ns)<br>0.296(ns)	Figure 7D<br>Figure 7D
Control (RNAi)	N2; *pL4440* (110)	13.07	-	22	-	-	-	Figures 7B, 7C, and 7F
	N2; *pL4440* (110)	14.28	-	20	-	-	-	Figures 7B, 7C, and 7F

	N2; *pL4440* (110)	13.29	-	20	-	-	-	Figures 7B, 7C, and 7F
100 mM glucose (RNAi)	N2; *pL4440* (110)	9.39	-28.16 [c]	12	-45.45 [c]	39.05[c]	<0.0001	Figures 7B, 7C, and 7F
	N2; *pL4440* (110)	10.23	-28.36 [c]	13	-35.00 [c]	44.05[c]	<0.0001	Figures 7B, 7C, and 7F
	N2; *pL4440* (110)	9.01	-36.90 [c]	12	-40.00 [c]	38.27[c]	<0.0001	Figures 7B, 7C, and 7F
Control (RNAi)	N2; *sbp-1* (110)	11.72	-10.33 [c]	19	-13.64 [c]	1.65[c]	0.199 (ns)	Figure 7F
	N2; *sbp-1* (110)	12.01	-15.90 [c]	19	-5.00 [c]	0.34[c]	0.478 (ns)	Figure 7F
	N2; *sbp-1* (110)	11.19	-21.64 [c]	18	-10.00 [c]	1.89[c]	0.290 (ns)	Figure 7F
100 mM glucose (RNAi)	N2; *sbp-1* (110)	9.42	-27.93 [c]	15	-31.82 [c]	10.31[c]<br>0.38 [d]	0.004<br>0.900(ns)	Figure 7F
	N2; *sbp-1* (110)	10.13	-29.06 [c]	13	-35.00 [c]	16.70[c]<br>0.47 [d]	0.004<br>0.123 (ns)	Figure 7F<br>Figure 7F
	N2; *sbp-1* (110)	10.46	-26.75 [c]	15	-35.00 [c]	18.27[c]<br>0.44 [d]	0.002<br>0.110(ns)	Figure 7F<br>Figure 7F
Control (RNAi)	N2; *hif-1* (110)	15.59	+19.28 [c]	22	0 [c]	95.16[c]	<0.0001	Figure 7B
	N2; *hif-1* (110)	18.20	+27.45 [c]	22	+10.00 [c]	98.23 [c]	<0.0001	Figure 7B
	N2; *hif-1* (110)	17.34	+21.43 [c]	20	0 [c]	89.60 [c]	<0.0001	Figure 7B
100 mM glucose (RNAi)	N2; *hif-1* (110)	14.96	+14.46 [c]	22	0 [c]	4.6[c]<br>0.79 [d]	0.065 (ns)<br>0.987 (ns)	Figure 7B
	N2; *hif-1* (110)	16.2	+13.45 [c]	21	+5.00 [c]	6.41[c]<br>0.620[d]	0.045<br>0.746 (ns)	Figure 7B<br>Figure 7B
	N2; *hif-1* (110)	15.87	+11.13 [c]	20	0 [c]	5.98[c]<br>0.521[d]	0.050<br>0.601 (ns)	Figure 7B<br>Figure 7B
Control (RNAi)	N2; *crh-1* (110)	19.14	+46.44 [c]	28	+27.27 [c]	40.62 [c]	<0.0001	Figure 7C
	N2; *crh-1* (110)	20.5	+43.56 [c]	29	+45.00 [c]	63.85 [c]	<0.0001	Figure 7C
	N2; *crh-1* (110)	21.2	+48.46 [c]	27	+35.00 [c]	52.14 [c]	<0.0001	Figure 7C
100 mM glucose (RNAi)	N2; *crh-1* (110)	17.77	+35.96 [c]	26	+18.18 [c]	21.58 [c]<br>3.8[d]	<0.0001<br>0.512 (ns)	Figure 7C
	N2; *crh-1* (110)	19.33	+35.36 [c]	24	+20.00 [c]	19.76 [c]<br>3.22[d]	<0.0001<br>0.443 (ns)	Figure 7C<br>Figure 7C
	N2; *crh-1* (110)	18.27	+27.94 [c]	25	+25.00 [c]	26.44 [c]<br>2.61[d]	<0.0001<br>0.347 (ns)	Figure 7C<br>Figure 7C

[Δ]: Throughout S2 Table, % change in lifespan and *P* values were calculated against the control condition. Increase (+) or decrease (-) in lifespan was indicated. (n) : number of worms, (ns) : not statistically significant, χ^2 *:* chi-squared.

[a] compared to wild-type animals in the control condition.
[b] compared to the same strain in the control condition against the HG condition.
[c] compared to N2 with empty vector pL4440 in the control condition.
[d] compared to the same RNAi in the control condition against the HG condition.
